# Evaluation of the efficacy of transarterial chemoembolization combined with microwave ablation followed by adjuvant therapy in patients with hepatocellular carcinoma

**DOI:** 10.3389/fimmu.2024.1337396

**Published:** 2024-02-06

**Authors:** Bowen Men, Huzhe Cui, Zhezhu Han, Xiuying Jin, Qiang Xu, Yongmin Jin, Zhengri Piao, Songnan Zhang

**Affiliations:** ^1^ Department of Oncology, Yanbian University Hospital, Yanji, China; ^2^ Department of Radiology, Yanbian University Hospital, Yanji, China; ^3^ Department of Radiation Oncology, Yanbian University Hospital, Yanji, China

**Keywords:** hepatocellular carcinoma, microwave ablation, transarterial chemoembolization, adjuvant therapy, lenvatinib, PD-1 antibodies

## Abstract

**Objective:**

This study aimed to explore the efficacy of transarterial chemoembolization (TACE) combined with microwave ablation (MWA) adjuvant to lenvatinib and anti–PD–1 antibodies for patients with hepatocellular carcinoma (HCC).

**Methods:**

A retrospective analysis of 67 patients with HCC treated at our hospital between October 2018 and May 2022 was conducted. All patients underwent a combination of TACE and MWA. Among them, 29 received postoperative treatment with molecular–targeted agents, like lenvatinib, along with anti–PD–1 antibodies such as sindilizumab, karelizumab, or tirilizumab. The remaining 38 patients did not receive postoperative systemic therapies, like targeted or immunotherapy. The survival and prognosis of all patients were analyzed.

**Results:**

Nine patients in the observation group and 29 patients in the control group experienced recurrence, and the median progression–free survival 1 (PFS1) was not reached ‘Not Applicable’(NA) and 17.05 months (P=0.035), respectively. Failure to combine adjuvant therapy was identified as an independent risk factor for tumor recurrence, and the observation group had a 0.245 times lower risk of recurrence compared to that in the control group (P=0.005). Multivariable Cox regression analysis confirmed that the maximum tumor size, and tumor number were risk factors for tumor recurrence. Patients with a large maximum tumor size had a 1.519 times higher risk of recurrence compared to those with a small maximum tumor size (P=0.006), and patients with a large number of tumors had a 5.978 times higher risk of recurrence compared to those with a small number of tumors (P=0.02). The median PFS2 of the two groups was 11.795 and 21.257 months, respectively, though not statistically significant (P=0.955). However, there was a disparity in the percentage of BCLC stages associated with recurrence between the two groups. In the observation group approximately 22.22% of patients progressed to stage C, while in the control group, this proportion was 34.48%. The observation group exhibited a lower risk of distant metastasis compared to the control group.

**Conclusion:**

Adjuvant treatment of HCC following TACE combined with MWA improved PFS and achieved better clinical outcomes compared to that with TACE combined with MWA alone.

## Introduction

The ablation of liver tumors is a radical treatment commonly used for early hepatocellular carcinoma (HCC), in addition to surgical resection and liver transplantation (1). Studies have shown that combining TACE with ablation for a single tumor ≤5 cm in diameter or 2–3 tumors ≤3 cm in diameter is superior to ablation alone (2–4). Ablation has a 5–year survival rate of 40%–70%, comparable to that of surgical resection (5). However, despite radical treatment, the recurrence rate remains high. A retrospective study revealed that the 1–, 2–, and 3–year recurrence rates of TACE combined with MWA were 47.8%, 78.3%, and 94.6% (6), respectively, possibly linked to microsatellite foci or microvascular invasion (MVI) around the tumor (7). Therefore, simple radical treatment cannot prevent tumor recurrence, and the adoption of effective adjuvant therapy is the key to reducing the recurrence rate and improving patient prognosis.

Lenvatinib and immune checkpoint inhibitors (ICIs) have been approved for use in patients with advanced unresectable HCC and are now widely used in clinical practice (8–10). Recent studies indicate that adjuvant lenvatinib following hepatectomy improves the early RFS rate of HCC patients with MVI by approximately 17.1%–27.3% (11), while adjuvant PD–1 antibodies improves the early RFS by approximately 22.8%–24.4% in HCC patients who have a combination of high–risk factors for recurrence after hepatectomy, such as portal vein tumor thrombus or tumor size of ≥5 cm (12). However, there is no consensus on the use of tyrosine kinase inhibitors (TKIs) and PD–1 antibodies as adjuvant therapies after resection or ablation of HCC. Few studies have reported the combination of TKIs and PD–1 antibodies after radical treatment. Therefore, we explored whether adjuvant therapy in this new combined modality could offer a survival benefit to patients.

In this study, a retrospective analysis of clinical patients was conducted. Patients with HCC who received postoperative adjuvant therapy were compared to evaluate the prognosis and efficacy of TACE combined with MWA followed by adjuvant targeted therapy and immunotherapy.

## Materials and methods

### Patient selection

Information on patients with early–stage HCC who underwent TACE combined with MWA between October 2018 and May 2022 at our hospital was collected. The inclusion criteria were as follows: (1) age ≥ 18 years; (2) pathologically diagnosed with HCC; (3) BCLC stage 0, A, or B1; and (4) Child-Pugh score ≤7. The exclusion criteria were as follows: (1) previous anti–tumor therapy (including molecular–targeted therapy or immunotherapy); (2) a history of other malignant tumors, associatedsevere organic dysfunction; and (3) incomplete clinical data.

### Treatment methods

TACE was performed using a Siemens Artis Zee digital subtraction angiography device for guidance, along with the Hengrui Medical RAPIDTHRUO microcatheter guidewire system. Embolization was performed using epirubicin 20mg, iodinated oil 5-10ml, and small amount of polyethanol embolization microspheres (white). MWA was performed within 1–4 weeks after the TACE treatment, and the MWA procedure was guided by ultrasound (US) using the Myriad Medical Devices system and computed tomography (CT) with the Aquilion One Toshiba Medical Systems. The TACE and MWA procedures were performed using the AnMTC–3C microwave ablation system (Vison Medicine). This system operates at a microwave transmitting frequency of 2450 ± 50 MHz, with a continuous wave output power ranging from 5 W–120 W. It employs, an ablation antenna with a length of 18 cm and a diameter of 2 mm. According to the size of each lesion to determine the ablation mode, ablation power and ablation time, the general ablation power of 50-70W, ablation time of 5-10 minutes, the ablation range of at least more than the tumor border 0.5-1.0cm. Experienced senior physicians conducted the TACE and MWA procedures.

The observation group received treatment with lenvatinib and PD–1 antibodies within 1 week after MWA. Lenvatinib was administered orally at a dose of 8 mg once daily. PD–1 antibodies (sindilizumab, karelizumab, or tirilizumab) were administered intravenously at a dose of 200 mg every 3 weeks. Patients were discontinued and switched to second–line therapy if treatment was not continued for more than two years or if disease progression occurred. Lenvatinib or PD–1 antibody therapy was discontinued in the event of grade 3 or 4 hematologic toxicity, dermatotoxicity, gastrointestinal toxicity, hypertension, or hepatic dysfunction, as defined by the Common Terminology Criteria for Adverse Events until the adverse reaction was relieved or resolved, evaluation by the attending physician as to whether to continue treatment with the original regimen.

### Follow–Up and evaluation

The study had a four–year follow–up with the cutoff date set for June 14, 2023. The primary endpoint of the study was progression–free survival (PFS1, PFS2). PFS1 was defined as the time from receiving MWA to the first disease recurrence. PFS2 was defined as the time from the date of treatment to the second occurrence of disease progression after the first disease recurrence. Enhanced CT or magnetic resonance imaging (MRI) of the liver was performed approximately one month after microwave ablation. CT or MRI scans were reviewed at 3–month intervals for 2 years after ablation and at 6–month intervals thereafter. In this study, recurrence was defined to include local progression and distant metastases. Local progression was defined as the presence of any new tumor lesions in the liver, whereas distant metastasis was defined as the presence of portal vein invasion, lymph node involvement, or tumor lesions in other organs.

### Data analysis

Statistical analysis was performed using SPSS 25.0. For baseline quantitative information, the mean and standard deviation were used to describe the data distribution if it conformed to normal distribution. The two independent samples t–test was used to compare the differences between the two groups. If the data did not confirm to a normal distribution, the data distribution was described using the median and minimum–maximum values and the difference between the two groups were compared using the Wilcoxon rank sum test. Categorical data were compared between the two groups using the chi–square test or Fisher’s exact probability method.

## Result

### Patient baselines

In this study, 90 patients with early–stage HCC who received TACE combined with MWA were screened according to the entry–exit–exclusion criteria. After excluding 23 cases (14 with an insufficient number of medication cycles and 9 lost to follow–up). 67 patients were finally included. Of these, 29 patients (observation group) received targeted immunotherapy following MWA and 38 patients (control group) received TACE combined with MWA without adjuvant therapy. The differences in gender, age, AFP, ALB, TB, ALBI grade, maximum tumor size, tumor number, hepatitis, ascites, Child–Pugh grade, and BCLC stage were not statistically significant (P>0.05), indicating a balanced baseline and comparability between the two groups (**Table 1**).

**Table 1:** Baseline Characteristics of Patient.

Variable	Observation Group	Control Group	Statistical volume	*P*
Gender				
Male	22	26	1.462	0.357
Female	7	12		
Age (years), Mean ± sd	67.07 ± 6.654	64.45 ± 9.489	1.328	0.189
AFP, Median (Min, Max)	10.47(1.4, 1200)	7.23(1.86, 9523)	-1.012	0.311
ALB, Median (Min, Max)	45 (28, 49)	44.5 (24, 53)	-0.381	0.703
TB, Median (Min, Max)	15.6(6.2, 33.1)	14.4(6.9, 57.2)	-0.202	0.840
ALBI grade, Median (Min, Max)	-3.04(-3.5, -1.46)	-2.98(-3.66, -1.36)	-0.443	0.658
Maximum tumor size, Median (Min, Max)	2.8(1, 8.5)	2.45(1.2, 6.4)	-0.634	0.526
Tumor number				
Single	21	25	3.077	0.215
Multiple	8	13		
Hepatitis				
Yes	26	35	<0.001	>0.999
NO	3	3		
Ascites				
YES	0	4	1.642	0.200
NO	29	34		
Child-Pugh grade				
A	29	35	0.906	0.341
B	0	3		
BCLC stage				
0	6	6	0.340	0.844
A	20	27		
B1	3	5		

AFP, alpha-fetal protein; ALB, albumin; TB, total bilirubin; ALBI, albumin-bilirubin; BCLC, Barcelona clinic liver cancer.

### PFS1 comparison

**Figure 1:**
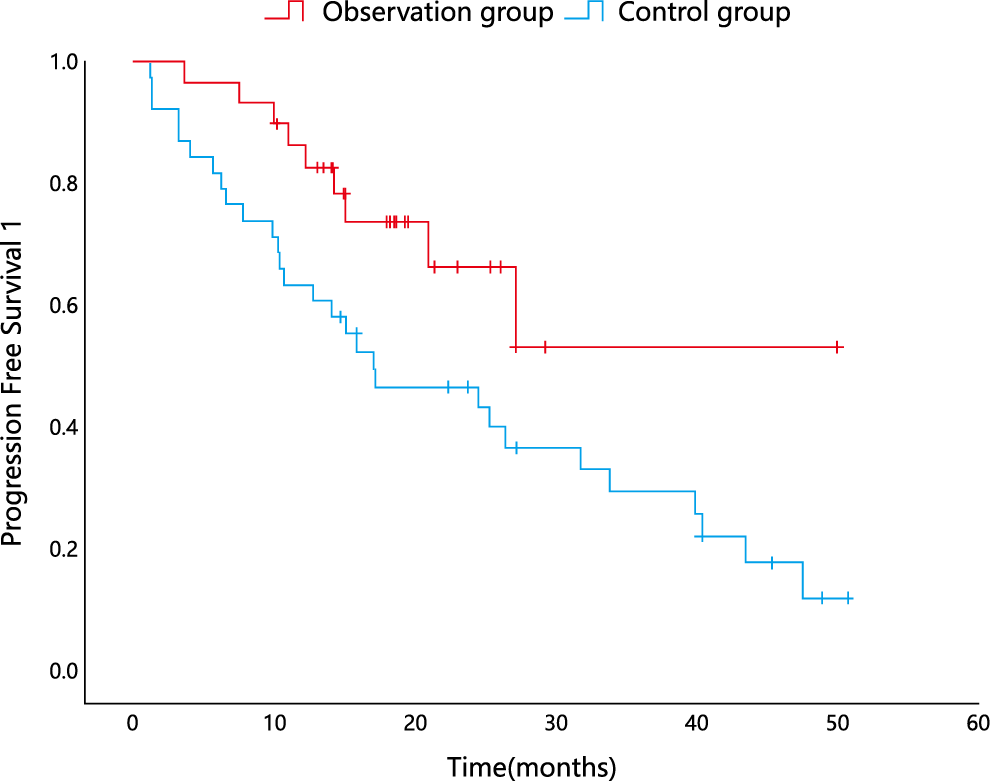
Kaplan–Meier analysis for PFS1.

Recurrence was observed in 9 patients in the observation group and 29 in the control group, with a median PFS1 of ‘NA’ (as no more than half of the patients in the observation group experienced disease recurrence) and 17.05 months, respectively. The difference in PFS1 between the two groups was statistically significant (P=0.035) using the log–rank test (**Figure 1**).

**Figure 2:**
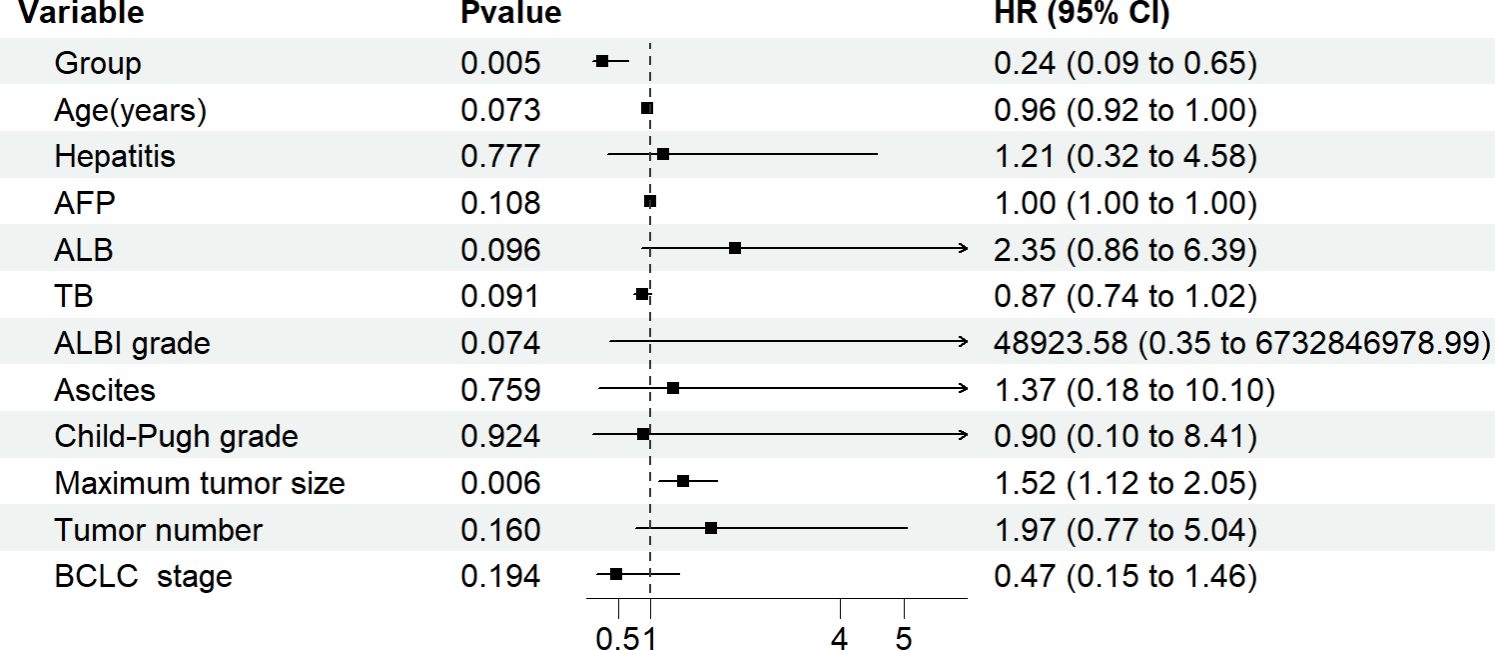
PFS1 multivariate analysis forest plot.

Multivariable Cox regression analysis was performed, including group, age, hepatitis, AFP, ALB, TB, ALBI grade, ascites, maximum tumor size, tumor number, BCLC stage, and Child-Pugh grade. In the analysis, the group and maximum tumor size were found to be statistically significant, while the other factors did not show a statistically significant effect on PFS1. The grouping factor (observation group=1, control group=0) exhibited a negative correlation with tumor recurrence (correlation coefficient=–1.407). After controlling for the factor of maximum tumor size, the effect of adjuvant therapy on PFS1 remained statistically significant. The observation group had a 0.245 times lower risk of recurrence compared to that of the control group (P=0.005). The maximum tumor size was positively correlated with tumor recurrence (correlation coefficient, 0.418), and the risk of recurrence in patients with a large maximum size was 1.519 times higher than that in patients with a smaller maximum size (P=0.006) (**Table 2**; **Figure 2**).

**Table 2:** Multivariate analysis of factors associated with PFS1.

Variable	B	SEx	χ	df	*P*	HR	95%CI
Group	-1.407	0.5	7.918	1	0.005	0.245	0.092-0.652
Age(years)	-0.041	0.023	3.208	1	0.073	0.96	0.917-1.004
Hepatitis	0.192	0.678	0.08	1	0.777	1.211	0.321-4.579
AFP	0	0	2.576	1	0.108	1	1
ALB	0.852	0.512	2.772	1	0.096	2.345	0.86-6.392
TB	-0.136	0.081	2.86	1	0.091	0.873	0.745-1.022
ALBI grade	10.798	6.037	3.199	1	0.074	4.89E+04	0.355-6.73E+09
Ascites	0.313	1.02	0.094	1	0.759	1.368	0.185-10.1
Child-Pugh grade	-0.108	1.142	0.009	1	0.924	0.897	0.096-8.413
Maximum tumor size	0.418	0.154	7.409	1	0.006	1.519	1.124-2.052
Tumor number	0.676	0.481	1.975	1	0.16	1.966	0.766-5.043
BCLC stage	-0.747	0.575	1.687	1	0.194	0.474	0.153-1.463

B, regression coefficient; SEx, standard error; χ2, chi square; df, degree of freedom; HR, Hazard Ratio; CI, confidence interval; AFP, alpha-fetal protein; ALB, albumin; TB, total bilirubin; ALBI, albumin-bilirubin; BCLC, Barcelona clinic liver cancer.

### PFS2 comparison

**Figure 3:**
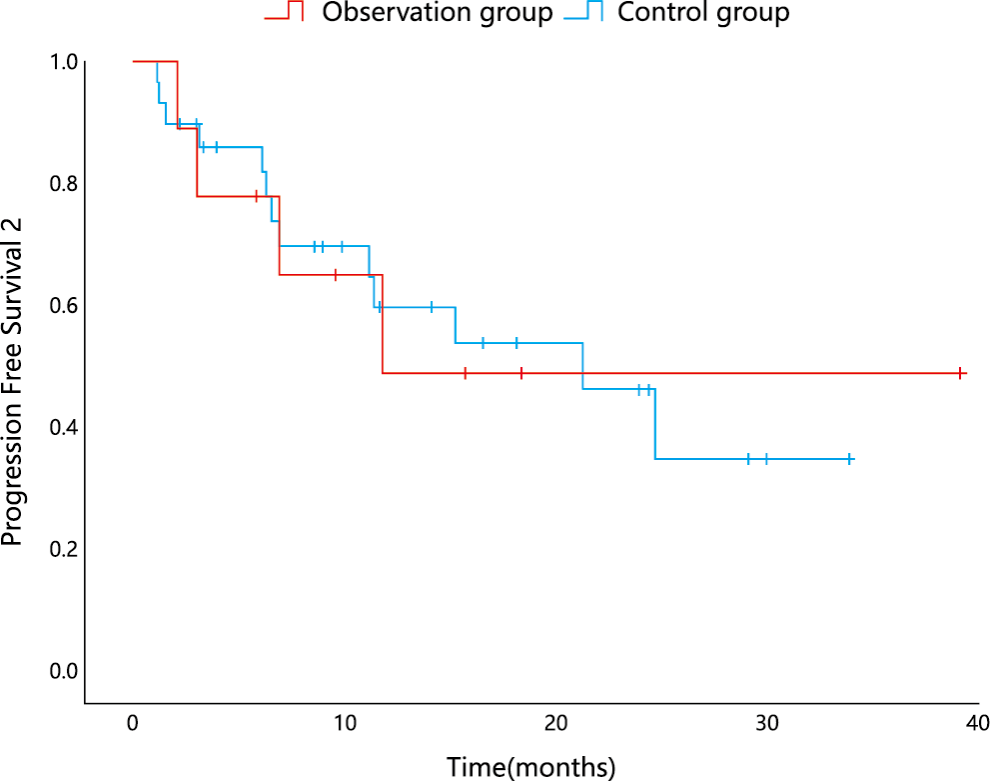
Kaplan–Meier analysis for PFS2.

The observation group had 4 patients with recurrence while 13 patients recurred in the control group, with a median PFS2 of 11.795 and 21.257 months, respectively. The difference in PFS2 between the two groups was not statistically significant (P=0.955) according to the log–rank test (**Figure 3**).

Using multivariable Cox regression analysis, which included group, age, hepatitis, AFP, ALB, TB, ALBI grade, ascites, maximum tumor size, tumor number, BCLC stage, and Child–Pugh grade, as well as variables related to the size of intrahepatic recurrent lesions after the first recurrence, number of intrahepatic recurrent lesions, extrahepatic metastases, vascular invasion, and BCLC stage after recurrence, the analysis revealed that only the tumor number was statistically significant. None of the other metrics, including group variables, had a statistically significant effect on PFS2. The tumor number was positively correlated with the second tumor recurrence (correlation coefficient=1.788). The risk of recurrence in patients with a large number of tumors was 5.978 times higher than that in patients with a small number of tumors (P=0.02) (**Table 3**).

**Table 3:** Multivariate analysis of factors associated with PFS2.

Variable	B	SEx	χ	df	*P*	HR	95%CI
Group	-0.307	1.083	0.08	1	0.777	0.735	0.088-6.146
Age(years)	-0.026	0.044	0.347	1	0.556	0.974	0.893-1.062
Hepatitis	-0.937	1.73	0.293	1	0.588	0.392	0.013-11.633
AFP	0	0	1.225	1	0.268	1	1-1.001
ALB	2.106	1.796	1.375	1	0.241	8.215	0.243-277.439
TB	-0.521	0.357	2.13	1	0.144	0.594	0.295-1.196
ALBI grade	25.664	21.372	1.442	1	0.23	1.40E+11	0-2.18E+29
Ascites	9.753	7.785	1.569	1	0.21	1.72E+04	0.004-7.29E+10
Child-Pugh grade	12.163	7.406	2.697	1	0.101	1.92E+05	0.095-3.86E+11
Maximum tumor size	0.34	0.244	1.943	1	0.163	1.405	0.871-2.266
Tumor number	1.788	0.77	5.396	1	0.02	5.978	1.322-27.025
Recurrent lesions size	0.575	0.315	3.335	1	0.068	1.778	0.959-3.295
Recurrent lesions number	0.196	0.249	0.623	1	0.43	1.217	0.747-1.982
Extrahepatic metastases	3.078	2.255	1.864	1	0.172	21.709	0.262-1801.743
Vascular invasion	0.541	1.859	0.085	1	0.771	1.717	0.045-65.603
Recurrent BCLC stage	-1.006	0.735	1.874	1	0.171	0.366	0.087-1.544

B, regression coefficient; SEx, standard error; χ2, chi square; df, degree of freedom; HR, Hazard Ratio; CI, confidence interval; AFP, alpha-fetal protein; ALB, albumin; TB, total bilirubin; ALBI, albumin-bilirubin; BCLC, Barcelona clinic liver cancer.

### Comparison of recurrence types

**Figure 4:**
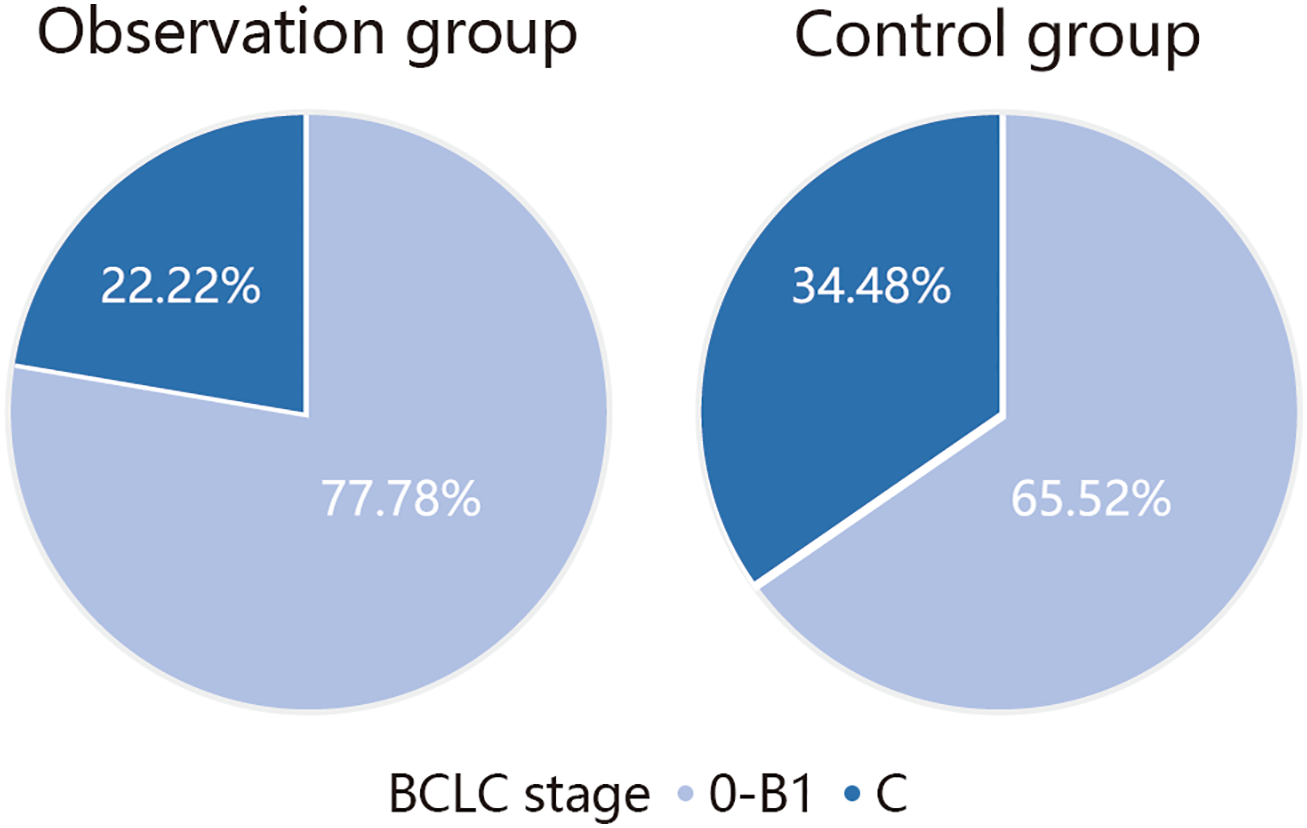
Comparison of recurrence types.

On analyzing the types of PFS2 recurrence, it was observed that seven patients with BCLC stage 0–B1 and two patients with stage C recurrence occurred in the observation group, while 19 patients with BCLC stage 0–B1 and 10 patients with stage C recurrence occurred in the control group. The number of patients who experienced recurrence in the observation group was lower than that in the control group, and the proportion of patients with stage C HCC in the observation group was lower than that in the control group among the patients who experienced recurrence (**Figure 4**).

### Safety

In this study, the incidence of adverse events in the observation group was 65.52%, with no grade 4-5 adverse events, the common grade 1-2 adverse events included itching and rash in 12 (41.38%), hypertension in 10 (34.48%), abdominal pain and diarrhea in 9 (31.03%), fatigue in 9 (31.03%), increased AST or ALT in 8 (27.59%), dry skin in 6 (20.69%), decreased appetite in 5 (17.24%), stomatitis in 2 (6.90%), and grade 3 adverse events included abdominal pain and diarrhea in 2 (6.90%), rash in 1 (3.45%), fatigue in 1 (3.45%), dry skin in 1 (3.45%), and the symptoms improved after stopping symptomatic treatment for 4 weeks and continued to be treated with the original regimen (**Table 4**).

**Table 4:** Adverse events.

Adverse events	Observation group(n=29)	
	Grade 1-2 AEs	≥ Grade 3 AEs
Pruritus, Rash	41.38% (12)	3.45% (1)
Hypertension	34.48% (10)	0
Abdominal pain, Diarrhea	31.03% (9)	6.90% (2)
Fatigue	31.03% (9)	3.45% (1)
Increased AST or ALT	27.59% (8)	0
Dry skin	20.69% (6)	3.45% (1)
Decreased appetite	17.24% (5)	0
Stomatitis	6.90% (2)	0

AST, aspartate aminotransferase; ALT,alanine aminotransferase.

## Discussion

Previous studies have found that many microsatellite foci are present around liver tumors after radical ablation therapy. Furthermore, 19% of satellite nodules in HCC nodules with diameters of 3 cm or less could not be identified before treatment (13), and approximately 20% of the microsatellite foci were located beyond 1.0 cm from the edge of the HCC lesion (14). The conventional ablation zone typically extends approximately 0.5–1.0 cm beyond the tumor margin (15). Consequently, microsatellite lesions located >1 cm beyond the tumor margin that have not been eliminated still retain the potential for regrowth, which is the main reason for local tumor recurrence.

This study demonstrated that the absence of an adjuvant therapy following combination treatment was an independent risk factor for tumor recurrence. The observation group did not achieve a median PFS1 since less than half of the patients had progressed to disease recurrence, while the control group had a median PFS1 of 17.05 months. Patients treated with adjuvant lenvatinib and PD–1 antibodies had a relatively longer PFS and a significant advantage in reducing the recurrence rate compared to those treated with TACE–MWA alone. A single–arm phase 2 trial reported on 30 patients with HCC treated with TACE in combination with MWA or radio frequency ablation. This study showed a median RFS of 7 months for patients receiving radical therapy alone, whereas patients treated with adjuvant PD–1 antibodies did not reach a median RFS (16). Another study reported 57 cases treated with lenvatinib as adjuvant post–resection therapy in patients with hepatitis B–related HCC with comorbid MVI, which demonstrated a significant advantage in terms of a reduced recurrence rate compared to patients who did not receive lenvatinib. Patients treated with lenvatinib postoperatively had improved recurrence and survival rates at 1 and 2 years compared to patients who did not receive adjuvant therapy with lenvatinib (11). Thus, combination adjuvant therapy offers advantages over radical therapy alone in improving the prognosis of patients with early–stage HCC. Multivariable Cox regression analysis showed that the maximum tumor size was a risk factor for tumor recurrence. Previous studies have reported that the rates of MVI in tumors <2 cm, 2–4 cm, and >4 cm were 25%, 31%, and 50% (17), respectively. Therefore, the larger the tumor, the higher the probability of microsatellite foci around the tumor, the emergence of MVI, and the increased the likelihood of tumor recurrence. Although a radical treatment, TACE in combination with MWA fails to eradicate microsatellite foci that cannot be detected by imaging around the tumor tissue, and the combination of lenvatinib and PD–1 antibodies can eliminate microsatellite foci, thus enhancing the anti-tumor effect (12, 18).

Multivariable analysis showed that the tumor number was a risk factor for PFS2, regardless of whether adjuvant therapy was administered. Many studies have shown that the tumor number is closely related to tumor recurrence and long–term prognosis (19). In this study, we found that the number of patients with recurrence after receiving adjuvant therapy was low, and there was no statistically significant difference in PFS2. However, the percentage of recurrence in relation to the BCLC stage was different between the two groups. The percentage of patients who had no adjuvant therapy and developed BCLC stage C was approximately 34.48% higher compared to that of the observation group, thus losing the chance for radical treatment. The risk of distant metastasis in patients who received adjuvant therapy was lower than that in patients treated with TACE–MWA alone. Most of the recurrent tumors were still early–stage HCC and hence had a better prognosis, a finding that holds great clinical significance.

The results of a large–sample randomized controlled trial (STORM) showed that sorafenib had no significant advantage in terms of recurrence–free survival (RFS) or overall survival in patients with early–stage HCC after surgical resection or ablative radical surgery (20). In addition, other studies, examining the efficacy and safety of adjuvant treatments, such as interferon therapy, vitamin K2, and systemic chemotherapy, in preventing HCC recurrence have not yielded positive results (21–23). A mouse model study found that local ablation combined with treatment with PD-1 antibodies significantly enhanced the immune response of tumor antigen-specific T cells, increased the intra-tumor Teffs/Tregs ratio, and induced a Th1-type anti-tumor immune response (24, 25), which resulted in prolonged disease control and inhibition of tumor recurrence and distant metastasis. It has been shown that the combination of TKIs and ICIs improves the prognosis of advanced HCC and is a commonly used treatment for recurrent HCC (8, 26). In a mouse model of HCC, clinical data indicated that the combination of lenvatinib and PD-1 antibodies had a better anti–tumor effect than either therapy alone (27). It has been observed that lenvatinib inhibits the angiogenic factor in the tumor microenvironment and blocks fibroblast growth factor receptor 4 (FGFR4). This reduces tumor PD–L1 expression and impedes the differentiation of regulatory T cells (Tregs), thereby improving the efficacy of PD–1 antibodies (28, 29). The combination of TKIs and PD–1 antibodies presents a favorable therapeutic option for eliminating microsatellite foci that have not yet been detected by imaging (30). This can compensate for the lack of eradication of residual cancer that remains after radical treatment. Therefore, we believe that the combination of adjuvant therapies can improve PFS and reduce the recurrence rate in patients with early–stage HCC compared with TACE–MWA alone.

In terms of safety, although the incidence of relevant adverse events in the treatment was not low, only grade 1–3 adverse events were observed which were manageable and well tolerated by the patients. This study has some limitations. First, being a retrospective study, it may have been subject to a selection bias in identifying patients who received adjuvant therapy. Second, the lack of statistical significance in PFS2 can be attributed to the relatively small sample size. Further analysis with a larger sample and extended follow–up duration is necessary to determine whether adjuvant therapy leads to different types of recurrence and yields greater clinical significance, thus presenting a new avenue for exploration. CONCLUSION: The preliminary observation of this study was that TACE combined with MWA, followed by adjuvant lenvatinib and PD–1 antibodies for HCC showed significant clinical benefits.

## Data availability statement

The original contributions presented in the study are included in the article/supplementary material. Further inquiries can be directed to the corresponding author.

## Ethics statement

The studies involving humans were approved by the Ethics Committee of Affiliated Hospital of Yanbian University. The studies were conducted in accordance with the local legislation and institutional requirements. The participants provided their written informed consent to participate in this study.

## Author contributions

BM: Data curation, Formal analysis, Investigation, Methodology, Resources, Validation, Writing – original draft, Writing – review & editing. HC: Methodology, Software, Validation, Writing – review & editing. ZH: Resources, Supervision, Writing – review & editing. XJ: Supervision, Validation, Writing – review & editing. QX: Formal analysis, Validation, Writing – review & editing. YJ: Formal analysis, Software, Supervision, Writing – review & editing. ZP: Funding acquisition, Writing – review & editing. SZ: Conceptualization, Data curation, Investigation, Project administration, Resources, Software, Supervision, Validation, Visualization, Writing – original draft, Writing – review & editing.
